# Chirped pulse Raman amplification in warm plasma: towards controlling saturation

**DOI:** 10.1038/srep13333

**Published:** 2015-08-20

**Authors:** X. Yang, G. Vieux, E. Brunetti, B. Ersfeld, J. P. Farmer, M. S. Hur, R. C. Issac, G. Raj, S. M. Wiggins, G. H. Welsh, S. R. Yoffe, D. A. Jaroszynski

**Affiliations:** 1 University of Strathclyde, SUPA, Department of Physics, Glasgow G4 0NG, United Kingdom; 2 UNIST, Banyeon-ri 100. Ulju-gun, Ulsan 689-798, South Korea

## Abstract

Stimulated Raman backscattering in plasma is potentially an efficient method of amplifying laser pulses to reach exawatt powers because plasma is fully broken down and withstands extremely high electric fields. Plasma also has unique nonlinear optical properties that allow simultaneous compression of optical pulses to ultra-short durations. However, current measured efficiencies are limited to several percent. Here we investigate Raman amplification of short duration seed pulses with different chirp rates using a chirped pump pulse in a preformed plasma waveguide. We identify electron trapping and wavebreaking as the main saturation mechanisms, which lead to spectral broadening and gain saturation when the seed reaches several millijoules for durations of 10’s – 100’s fs for 250 ps, 800 nm chirped pump pulses. We show that this prevents access to the nonlinear regime and limits the efficiency, and interpret the experimental results using slowly-varying-amplitude, current-averaged particle-in-cell simulations. We also propose methods for achieving higher efficiencies.

High power, short pulse laser systems have become valuable tools with significant scientific and societal value^1^. Considerable international effort is being focused on developing the next generation of ultra-high power systems capable of realizing exawatt powers^2^. These systems are based on chirped pulse amplification (CPA)^3^ and utilise solid state media, but the next step is very challenging because the low damage threshold of optical elements results in very large amplifiers and compressors. This constraint has led to the suggestion of stimulated Raman backscattering (SRBS) in plasma^4^ as an alternative amplification method^5^ suitable for high field intensities, where energy is directly transferred from a pump beam to a short seed beam. SRBS amplification is achieved through the generation of a plasma density echelon, which acts as a transient grating that scatters the pump pulse into the seed, without requiring a temporally stretched seed pulse or storing the energy of the pump. Resonant excitation of the plasma wave occurs when *ω*_0_ = *ω*_1_ + *ω*_*p*_ and **k**_**0**_ = **k**_**1**_ + **k**_**p**_, where (*ω*_0_, **k**_**0**_), (*ω*_1_, **k**_**1**_), (*ω*_*p*_, **k**_**p**_) are the respective frequencies and wave-vectors of the pump, seed and plasma waves. SRBS has potential to significantly reduce the size and cost of laser systems, however, its feasibility has not yet been demonstrated and energy transfer efficiencies have been limited to a few percent for seed energies of a few mJ^6^,^7^,^8^,^9^,^10^,^11^,^12^,^13^,^14^, which contrasts with current theoretical predictions^5^,^15^,^16^,^17^,^18^. While plasma has no damage threshold, several phenomena can lead to the saturation of the gain. In the fluid regime, where a wave description is sufficient to describe the interaction, electrostatic decay instability and/or modulational instability are important^19^. In the kinetic regime, where wave-particle interaction becomes important, the dominant saturation mechanisms are detuning due to the Bohm-Gross shift^20^, wavebreaking and particle trapping^21^/nonlinear Landau damping, which arise from the plasma finite temperature, as will be discussed in this paper. Control of the plasma kinetic behaviour is crucial for the development of an efficient amplifier and, therefore, an in-depth understanding of these physical processes is required.

While significant progress has been made in this direction^14^,^22^,^23^,^24^,^25^,^26^,^27^, here we provide a comprehensive investigation of the saturation processes supported by experimental and numerical results in long, homogeneous plasma channel. Raman amplification is experimentally studied in a hydrogen-filled capillary discharge waveguide^28^ using a long duration, frequency chirped pump, which leads to a spatio-temporal distribution of the gain that enables amplification of the whole seed spectrum^13^,^29^. In addition, the chirped pump helps reduce spontaneous Raman scattering^16^,^30^ and is useful for counteracting the time dependent increase in the plasma frequency due to heating^31^. The experiments have been carried out using the TOPS Ti:sapphire laser system^32^; details of the experimental setup can be found in the Supplementary Information section. The pump is a ~250 ps duration chirped pulse, with a central wavelength of 800 nm and an energy of 1 J. The seed pulse has a minimum duration of 70 fs, an energy of up to ~2 mJ, and a central wavelength of 817 nm, apart from one set of data obtained at low pump power in Fig. 1 where the central wavelength is 836 nm (see Methods and Supplementary Information). The two beams are focused to a waist of ~60 μm onto opposite sides of a 4 cm long, 300 μm diameter plasma-filled hydrogen discharge capillary waveguide. This enables interaction over the full duration of the pump in uniform plasma densities of 1–2 × 10^18^ cm^−3^. After interaction, the seed pulse properties are measured using three diagnostic systems: i) an imaging system consisting of a 4× microscope objective and a 12 bit CCD camera (Point Grey, Flea2), which is used to measure the relative energy gain, ii) a spectrometer (OceanOptics, USB4000) connected to an optical fiber to observe spectral changes, and iii) a frequency resolved optical gating (FROG) based diagnostic system, which monitors phase and temporal profile modifications. Consistent series of measurements have been taken for various seed and pump energies, seed durations and chirps. The main results from three typical runs are summarized in Fig. 1, where seed energies range from 0.4 mJ to 2 mJ, seed durations from 75 fs (fully compressed) to 330 fs (positive chirp), and pump energies from 100 mJ to 880 mJ. Figure 1a presents the measured amplification factor, together with the theoretically expected amplification factor for both cold and warm (30 eV) plasma, as function of pump power. The theoretical values are calculated making two observations: i) for a chirped pump, the amplification factor of the seed amplitude in the linear regime is given by *G* = exp(*πγ*^2^/2*α*)^13^,^29^ with the resonant energy transfer rate (growth rate in the small signal regime)^4^


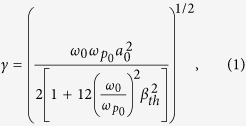


which has been extended to finite temperature plasma. *a*_0_ is the normalized vector potential of the pump, *α* is the pump linear frequency chirp rate defined as Δ*ω*/*τ* where Δ*ω* is the pump frequency bandwidth and *τ* its duration, both given as full width half maximum. 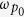 is the cold plasma frequency, and *β*_*th*_ is the electron thermal velocity normalized to *c*, the speed of light in vacuum; and ii) the pump power is halved to account for the fraction of energy concentrated in the beam waist (determined experimentally). The initial plasma temperature is 3–5 eV^33^, which is estimated to increase to ~30 eV towards the end of its propagation for our highest pump power, due to inverse bremsstrahlung heating. Apart from the run at low pump powers, resonance is only satisfied at the rear of the pump, where the plasma is warm, which is the main reason for gain reduction, as can be seen from equation (1). For pump powers up to 2.5 GW, the measured amplification factors are in good agreement with the linear Raman theory, for plasma temperature up to 30 eV. For higher powers (data points labeled A and B in Fig. 1a–c), the measured amplification factors, of up to ~7.5 for a 275 fs, 560 μJ seed (see Fig. 1d–f), are lower than expected and thus other saturation mechanisms must be identified.

Important information is obtained from studying the seed spectral characteristics after interaction. Figure 1b,c show that the spectral bandwidth and the mean wavelength change as function of 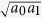, with *a*_1_ the seed normalized vector potential. To evaluate 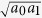 the initial peak amplitude of the pump and the maximum peak amplitude of the seed after interaction are used. This is chosen as an indication of the ponderomotive amplitude driving the plasma wave. For low values of 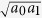, the spectral bandwidth of the seed initially reduces by 20%, suggesting spectral gain narrowing. But, as the values of 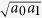 increases, the seed spectral bandwidth quickly broadens, up to +30%. In addition, the mean wavelength of the seed is pushed towards shorter wavelengths when 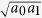 increases. As the plasma wave is driven more strongly, the seed spectra become broader, extending mainly on the high frequency side. This is attributed to particle trapping (or equivalently nonlinear Landau damping) and has been generally observed in several experiments, however such a scaling has never been presented. Particle trapping occurs when *k*_*p*_*λ*_*D*_ is large^34^, where *λ*_*D*_ is the Debye length. Because the wave traps electrons, the wave frequency decreases^35^. Wave-particle mechanisms are the principal nonlinear processes for driven plasma waves with *k*_*p*_*λ*_*D*_ > 0.29 (ref. 34). Under our experimental conditions the plasma temperature is 30 eV, at maximum pump power, *k*_*p*_*λ*_*D*_ ≥ 0.6 indicating operation in the kinetic regime, which is consistent with our experimental findings. Particle trapping is expected to play an important role in the saturation of SRBS. However, proper quantification has previously been difficult because existing models are only valid for *k*_*p*_*λ*_*D*_ ≤ 0.4 (ref. 36).

An important observation from our experimental studies is the indirect effect of the seed chirp on the amplification process. An experimental run has been conducted using different pump energies, and different seed durations with positive chirp (PC) and negative chirp (NC), respectively. The energy of the seed is maintained at 750 μJ. Figure 2 illustrates the results obtained for three different pump energies 230, 400 and 940 mJ and five seed durations from 400 fs, with a positive chirp, to 375 fs, with a negative chirp. Apart for the lowest pump energy where the plasma wave is not strongly excited, the use of fully compressed or PC seeds consistently results in a higher amplification factor, usually by a factor ~2. It has been theoretically shown that the sign of the seed chirp can be chosen to take advantage of the group velocity dispersion (GVD)^37^, but for our experimental parameters GVD is negligible and we attribute the difference in amplification to wavebreaking, which is understood as the maximum (or breaking) plasma wave amplitude for warm plasma. Coffey has shown, using a simplified model, that strong adiabatic heating rapidly limits the electrostatic field amplitude, which decreases as the ratio 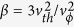 increases, where *v*_*th*_ is the electron thermal velocity and *v*_*ϕ*_ the phase velocity of the plasma wave^21^. The maximum field amplitude, *f*_*max*_, normalized to the cold limit value, is given by:


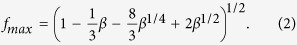


Due to the large seed chirp rate, the beat-wave excites a plasma wave with a rapidly varying phase velocity along the seed temporal envelope, with *v*_*ϕ*_ increasing (decreasing) for negative (positive) chirps respectively. As will be illustrated later, for our parameters, equation (2) shows that the plasma wave is heavily damped at the front of the negatively chirped seed because *v*_*ϕ*_ is lower than the thermal velocity. The plasma wave amplitude has therefore less time to grow, leading to saturation. In the case of the positively chirped seed, the plasma wave is excited initially from the front of the pulse, which extends the period of growth, even when damping of the plasma wave occurs at later times because of the reduction in *v*_*ϕ*_.

Our experimental results indicate that thermal effects play a key role in the SRBS saturation mechanism. Heating not only leads to a reduction in the growth rate but also to particle trapping that enhances saturation through the change in the plasma frequency and limitation of the plasma wave amplitude.

To obtain further insight into the dynamics of the amplification process, SRBS is numerically studied using the 1D slowly-varying-amplitude, current-averaged particle-in-cell code, aPIC^38^. The pump parameters are as follows: central wavelength 805 nm, bandwidth at FWHM 35 nm, duration 230 ps (flat profile, positive chirp). *a*_0_ values of 1.93 × 10^−3^, 2.73 × 10^−3^ and 3.86 × 10^−3^ are used, representing powers of 1, 2 and 4 GW, respectively. The seed spectra are centered at 817 nm and have a 10 nm FWHM bandwidth. The temporal profile is Gaussian with a FWHM of 275 fs and a positive chirp. A *a*_1_ of 2.86 × 10^−3^ (corresponding to ≈0.5 mJ) is used in conjunction with each pump power value and *a*_1_ of 5.72 × 10^−3^.(≈2 mJ) is used only for the highest *a*_0_. The plasma density is set to 1 × 10^18^ cm^−3^. aPIC is a collisionless code, therefore the time-dependent plasma temperature is evaluated beforehand from the ion-electron collision rate, and fresh plasma electrons enter the moving window with the corresponding thermal velocity distribution (see Supplementary Information). Because of *ω*_*p*_/*ω*_0_ ≪ 1 a dispersionless solver is used^38^, which has been previously checked to ensure that dispersion has no effect.

The simulation results are summarized in Fig. 1 together with the experimental results. The amplification factors are in good agreement with the theoretical values for warm plasma, except for the simulation with large *a*_1_. Spectral bandwidth narrowing followed by broadening as function of 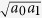 and spectral shifts towards high frequencies are also observed, which are consistent with measurements. Figure 3 illustrates, in detail, the amplification process for positively and negatively chirped seeds with *a*_1_ = 2.86 × 10^−3^, and a pump of highest intensity. Figure 3g,h show the amplification factor and mean wavelength shift as a function of time for PC and NC seeds, respectively. It is clearly observed that i) the amplification factor for the PC seed is three times larger than the amplification factor for the NC seed, confirming our experimental findings, ii) spectral shift towards high frequencies (indicating the onset of particle trapping) occurs when an amplification factor of 3–4 is reached, corresponding to the few mJ seeds for our parameters. To illustrate this, snapshots of the temporal and spectral profiles for PC and NC seeds at *t* = 0, 105 and 120 ps are shown in Fig. 3a,c and Fig. 3b,d, respectively. The difference in gain is explained by comparing, in Fig. 3e,f, the electron phase-space plots at *t* = 105 ps extracted at the front, peak and back of the pulse, and plotting them together with the corresponding electrostatic field amplitudes. In agreement with our argument using Coffey’s theory, the plasma wave amplitude is larger by more than a factor two for the PC case, when compared with the NC case. To be more quantitative, the calculated plasma wave velocity and maximum amplitude (from equation (2)) are plotting in Fig. 4c,d for positive and negative chirp respectively. A plasma wave with an amplitude of 20% of the cold limit amplitude can be excited from the front of the PC seed, while the plasma wave is suppressed when considering the negative chirp case. Also, particle trapping is clearly locally observed in Fig. 3e,f with the full electron phase space maps presented in Fig. 4a,b. Trapped electron fractions of up to 8% (PC) and 3% (NC) are obtained. Strong trapping is observed at the peak of the seed beam, with the fraction of trapped particles remaining constant at the back of the pulse. The fraction of trapped electrons increases by up to 15% for the run with *a*_1_ = 5.72 × 10^−3^, leading to a large plasma frequency shift that explains the drop in the amplification factor.

Reaching amplification factors beyond the limit governed by the observed saturation mechanisms requires operation in a regime where thermal effects are either compensated for or can be neglected. In the former case autoresonance occurs when the increase in the plasma density in the direction of propagation of the plasma wave partly (or fully) compensates the nonlinear frequency shift^39^. For the waves to become phase-locked a spatial parabolic density profile is required, which is experimentally difficult to achieve. However, a promising approach proposed by Malkin and Fisch^40^, is to amplify as close as possible to wavebreaking in the pump depletion regime and in the hydrodynamic limit. While their 3-wave interaction description includes the relativistic electron nonlinearity for determining the optimum amplification time before energy flow slows down or reverses, it does not take into account thermal effects as described in our paper, nor a pump chirp.

To establish whether growth can be maintained in the kinetic regime, 1D aPIC simulations, which include dispersion, have been performed with parameters obtained from the analytical formulae in ref. 40: *a*_0_ = 4.35 × 10^−3^ (4 × 10^13^ Wcm^−2^), pump central wavelength *λ*_0_ = 800 nm, pump duration *τ*_*p*_ = 300 ps, *a*_1_ = 0.01, seed central wavelength *λ*_1_ = 850 nm, seed duration *τ*_*s*_ = 50 fs. The plasma density is chosen to be 5 × 10^18^ cm^−3^ for an experiment to be feasible using a capillary or gas cell over an extended length. This has the advantage of avoiding Brillouin scattering and laser beam filamentation because the formation time *τ*_*fil*_ ≈ 60 ns ≫ *τ*_*p*,*s*_. The plasma temperature is set to ~5 eV at the beginning of interaction, increasing to ~85 eV when interaction finishes. From our parameter choice, the best attainable results are: a maximum seed intensity *I*_*max*_ = 9 × 10^16^ Wcm^−2^ (fluence ~4.5 kJ cm^−2^), a minimum seed duration *τ*_*min*_ = 50 fs for an optimum interaction time *t*_*M*_ = 85 ps. The plasma temperature increase results in a change in plasma frequency of up to 22% at *t*_*M*_, which is sufficient to avoid strong amplification from noise^40^. Table 1 summarizes the characteristics of the amplified seed obtained from different numerical studies, mainly comparing results between fluid and aPIC simulations, different temperatures and use of a chirped/unchirped pump. Intensities reached in the fluid/unchirped runs at 0, 25 and 50 eV show good agreement with the expected values, seemingly providing a robust amplification scheme because temperature changes do not strongly affect the seed output. In contrast, the seed intensity obtained in the aPIC simulation is half that expected from the 3-wave model. However, extending the interaction duration leads to continuous amplification to an intensity of 2.8 × 10^17^ Wcm^−2^ and pulse duration of 15 fs at time *t*_*F*_ = 147 ps. At this intensity, while longitudinal instabilities should be properly accounted for, transverse instabilities, that cannot be modeled here, would need to be considered. However, this still demonstrates that high energy transfer efficiencies should be possible. The use of a chirped pump is useful for avoiding Raman backward and forward amplification from noise and, as previously mentioned, to keep the three waves resonant by compensating for changes in the plasma frequency due to heating. Numerical studies with a positive pump chirp with Δ*ω*/*ω* of 1% and 3.8% (the value for our current experimental pump beam) have been performed. While for the former the gain is not drastically reduced in the aPIC simulation, for the latter, the gain reduction is appreciable (also when considering the fluid simulation). Figure 5 shows the seed at times *t*_*M*_ and *t*_*F*_ for each aPIC simulation and at time *t*_*M*_ for the fluid simulation with a monochromatic pump. Inset of Fig. 5 illustrates that the seed depletes the monochromatic pump at *t* = 136 ps.

In conclusion, we have experimentally studied SRBS saturation mechanisms in the kinetic regime and compared our results with 1D simulations. We find that the pump chirp and finite plasma temperature significantly reduce the amplification factor. Moreover, the electron thermal distribution not only affects the growth rate *γ*, but also leads to particle trapping, which further reduces the growth through saturation of the plasma wave amplitude and to a nonlinear frequency shift, which increases as 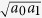 increases. To reduce this deleterious effect, it is necessary to choose *v*_*ϕ*_ ≫ *v*_*th*_, which requires high densities, which are susceptible to filamentation. To numerically find a promising amplification regime we have followed Malkin and Fisch^40^, and have selected a plasma density of 5 × 10^18^ cm^−3^, which can be relatively easily obtained in capillaries or gas cells to produce a long interaction region. In 1D, this scheme seems to be robust to heating, kinetic effects and small pump chirp. Seed intensities of ~3 × 10^17^ Wcm^−2^ and pulse compression could be achievable for very moderate pump intensities.

## Methods

### Experimental setup

The general schematic of the experimental set-up is presented in Supplementary Figure 1. The seed and pump originate from the same laser beam, therefore care must be taken to prevent transmission of the beams back into the laser chain. The initial beam polarization is horizontal and polarisers and quarter wave plates are used in each beam line to first change the polarization to circular in the region of interaction and, then, to vertical to reflect the beams out of the initial path after interaction, acting as a discriminator. Single-stack dielectric coated mirrors are used to minimize polarization changes after reflection.

### Gas-filled capillary discharge waveguide

The plasma channel waveguide is formed by ionizing H_2_ with a high voltage discharge formed by applying a potential difference of 15 kV across a pre-filled alumina capillary^41^. Thermal conduction results in an inhomogeneous temperature distribution. The plasma cools against the capillary wall to form a parabolic radial density distribution, with *n*(*r*) = *n*_0_ + Δ*n*(*r*/*r*_*m*_)^2^. *n*_0_ is the on-axis density, Δ*n* the density increase at the capillary wall and *r*_*m*_ is the capillary radius. The laser beam couples to the fundamental mode of the waveguide provided the beam waist (radius at 1/*e*^2^ in intensity) 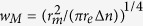, where *r*_*e*_ is the electron radius. An approximate value is given by 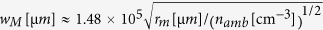, where *n*_*amb*_ is the ambient or unperturbed electron density. For a density of 1 × 10^18^ cm^−3^, *w*_*M*_ ≈ 57 μm. The capillary and electrodes are embedded in a perspex housing mounting on a 5-axis stage (linear XYZ stage for translation motion and a gimbal mount for rotation).

### White light generation

White light generation in a noble gas is used to either entirely shift the laser spectrum to lower frequencies or to broaden it before filtering out the higher frequencies. White light generation is due to filamentation in a gas. Argon is chosen as a good compromise between the requirement of a high ionization threshold and a large nonlinear refractive index. Supplementary Figure 2 presents the detailed experimental arrangement while supplementary Figure 3 gives an example of the optical characteristics of the seed. The main challenge in using a white light generator is reproducibility, which is also the reason for only using the compressor as a cut-off filter for most experiments.

### FROG technique

The amplitude and phase of probe pulses with and without amplification are measured using the second-harmonic generation frequency-resolved optical gating (SHG-FROG) technique^42^. The pulse to be characterised is mixed with a delayed replica in a nonlinear crystal. The up-converted signal is conveyed to an imaging spectrometer (Oriel MS127i) connected to a 8 bit CCD camera, which records the 2D spectrogram of the signal intensity versus wavelength and time delay. The amplitude and phase of the electric field is retrieved using commercial software from Swamp Optics^43^. Inversion of a SHG trace is ambiguous in the direction of time, and therefore the sign of the chirp cannot be determined. Therefore, an additional measurement is performed with the input beam passing through a glass window, to add positive dispersion, which changes the pulse duration. Thus, positively chirped pulses are stretched and negatively chirped pulses are compressed.

### Analysis of experimental results

For each measurement, ten data acquisitions are recorded with and without the pump. Because of fluctuations in measurements mainly due to laser pointing and capillary discharge jitter, for each data set, only the analysis of the single best acquisition for the amplified seed is kept. Extracted beam characteristics are compared with the averaged characteristics of the 3 best guided shots without pump. Standard deviation of the spectrum is used as a measure of the spectral bandwidth and the central wavelength is obtained through the weighted arithmetic mean.

### Simulations

aPIC is a 1-dimensional, averaged particle-in-cell (PIC) code developed for the simulation of Raman amplification in plasma^38^. It employs envelope equations for the two laser pulses and a particle description for the plasma, where motion on fast time scales is eliminated. The simulation time step does not resolve the laser period and, as a result, simulations are faster than a full PIC code by a factor of *ω*/*ω*_*p*_, where *ω* is the laser frequency. A moving window, which defines the simulation domain, is implemented to improve computational efficiency. Modifications have been made to the original code, which include implementation of chirped laser pulses, single-pulse ponderomotive contributions (which drive Raman forward scattering and wakefield generation), a dispersive model for the laser solver, and a time-dependent temperature function.

The aPIC numerical model is polarization independent, therefore, input values for the normalized vector potentials of the beams are given as root-mean-square (rms) values, such that 
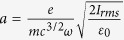
, with −*e* and *m* the electron charge and mass, *I*_*rms*_ the rms value of the beam intensity, *ε*_0_ the vacuum permittivity.

### Data availability

The data associated with this research is available at doi: 10.15129/a55d0576-fdff-4a0f-b0da-70e025523c7a.

## Additional Information

**How to cite this article**: Yang, X. *et al*. Chirped pulse Raman amplification in warm plasma: towards controlling saturation. *Sci. Rep*. **5**, 13333; doi: 10.1038/srep13333 (2015).

## Supplementary Material

Supplementary Information

## Figures and Tables

**Figure 1:**
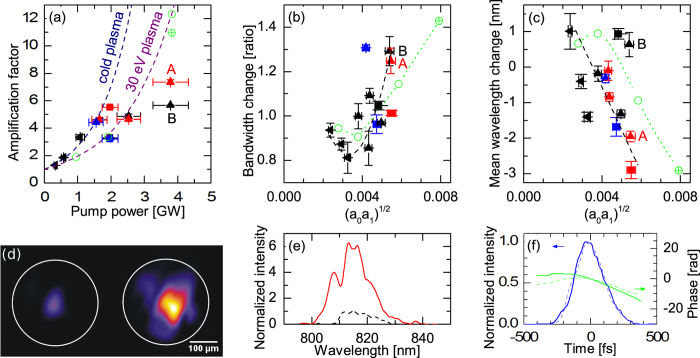
Main experimental results. Initial seed properties: (◀) 200 fs, 400 μJ, (■) 75 fs, 1 mJ, (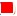) 110 fs, 2 mJ, (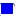) 140 fs, 2 mJ, (▲) 160 fs, 560 μJ, (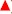) 275 fs, 560 μJ, (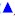) 330 fs, 560 μJ. Circles represent simulation outputs for direct comparison with experiment: (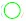) 275 fs, *a*_1_ = 2.86 × 10^−3^, (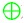) 275 fs, *a*_1_ = 5.72 × 10^−3^. (**a**) Measured amplification factor as function of pump power. The 2 theoretical lines are derived from Eq. (1). (**b**) Seed spectral bandwidth change as function of 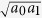. (**c**) Seed spectral mean wavelength change as function of 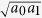. (**d**) Seed transverse profile images at the capillary output: (left) without pump, (right) with pump present. (**e**) Spectra related to images in (**d**). Dashed (black) line: initial spectrum, solid (red) line: spectrum after amplification. (**f**) FROG traces showing retrieved temporal profiles and phases. Dashed/solid (blue) line: temporal profile before/after interaction. Dashed/solid (green) line: phases before/after interaction. As shown in this measurement, seed duration is mainly conserved and increase in the spectral bandwidth leads to a larger chirp rate. In (**b**,**c**) dashed lines are guides for the eyes.

**Figure 2:**
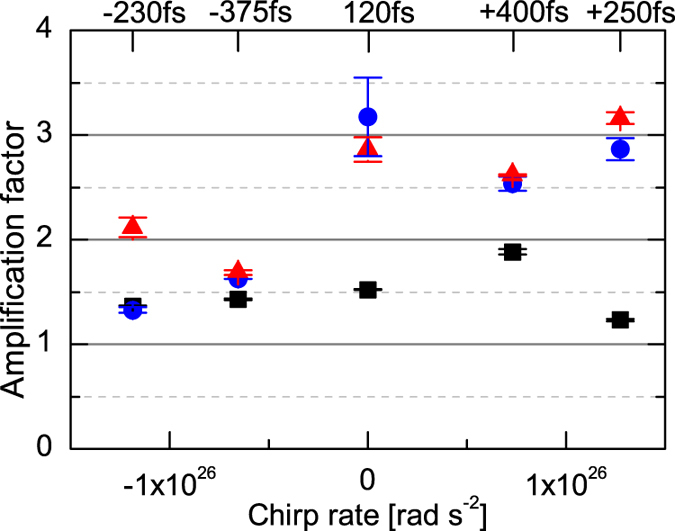
Amplification factor as function of seed chirp rate and pump energy: (■) 234 mJ, (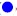) 400 mJ, (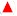) 944 mJ. The top scale indicates the seed duration for a given chirp rate.

**Figure 3:**
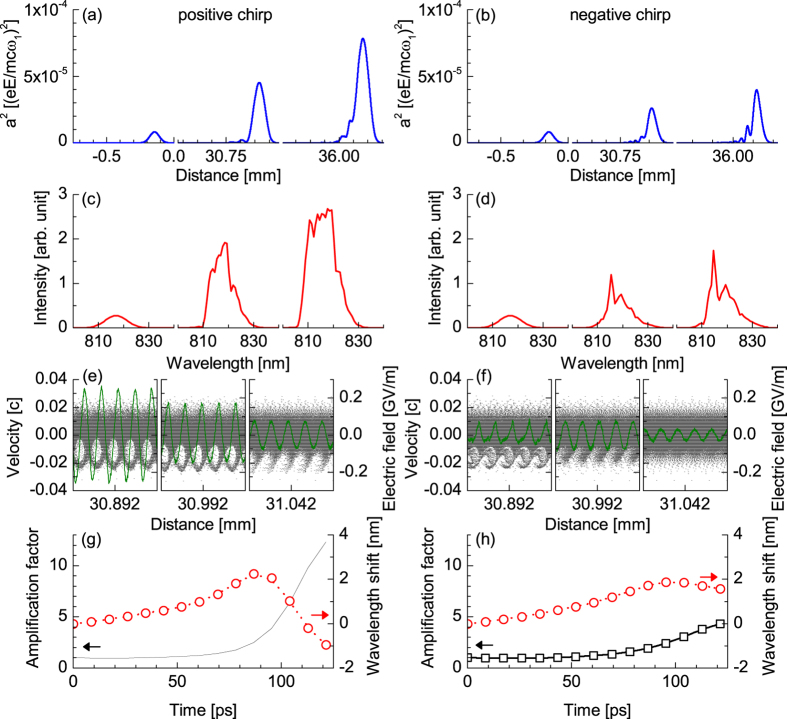
Numerical results for a positively (**a,c,e,g**) and negatively (**b,d,f,h**) chirped seed with *a*_1_ = 2.86 × 10^−3^ counter-propagating with the pump with *a*_0_ = 3.86 × 10^−3^. The seed is positioned at the front of the 750 µm long moving window and travels to the right in the laboratory frame. (**a**,**b**) temporal seed profiles at 0, 105 and 120 ps for PC/NC seed, (**c**,**d**) spectral seed intensities at 0, 105 and 120 ps for PC/NC seed, (**e**,**f**) electrostatic field amplitude and electron phase space extracted at time *t* = 105 ps and position 500, 600 and 650 *μ*m for PC/NC seed, (**g**,**h**) amplification factor (square) and mean spectral wavelength (circle) as function of interaction time for PC/NC seed.

**Figure 4:**
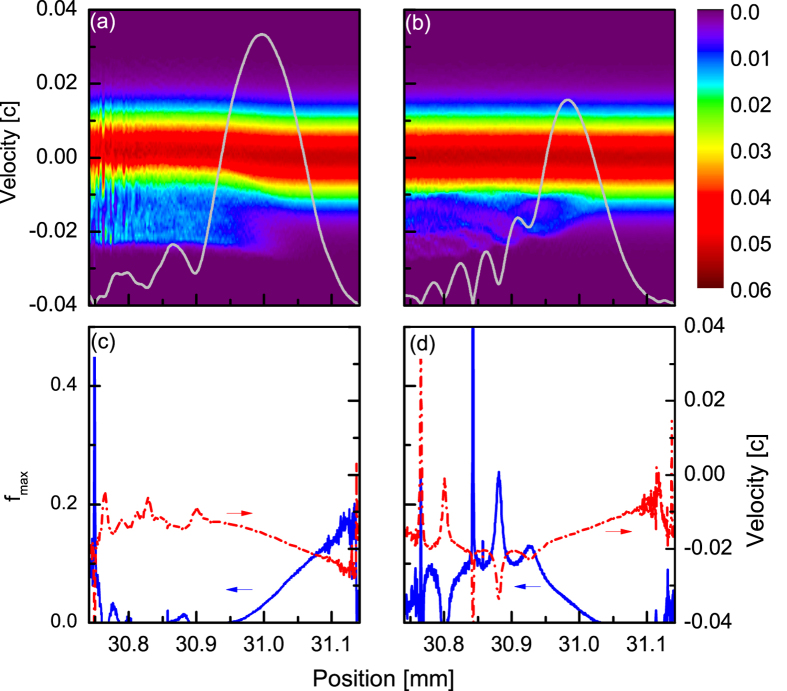
Electron thermal velocity distribution maps with superimposition of the seed amplitude (solid line) for (a) positive chirp, (b) negative chirp. The color scale indicates the ratio of electrons at a given position and velocity. Also presented, the calculation of the plasma wave velocity (dashed-dotted line) and maximum amplitude derived from equation (2) (solid line) for (**c**) positive chirp, (**d**) negative chirp.

**Figure 5:**
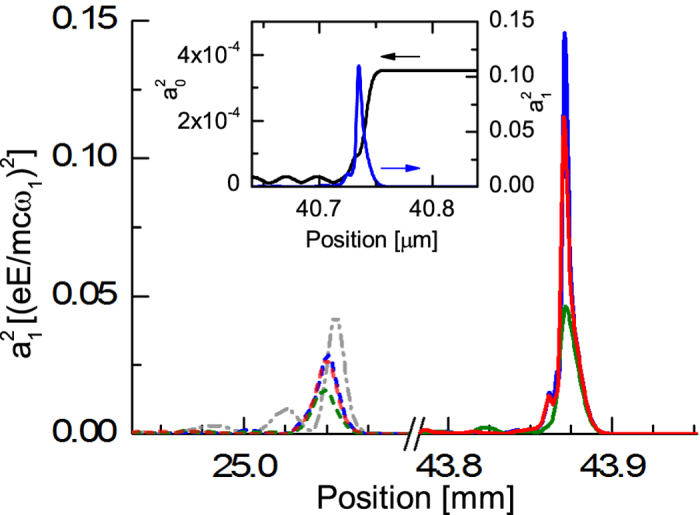
aPIC seed snapshots at *t* = *t*_*M*_ (dashed line) and *t* = *t*_*F*_ (solid line) for different pump bandwidths: (green) 3.8%, (red) 1%, (blue) quasi-monochromatic. The (gray) dashed-dot line represents the seed snapshot at *t* = *t*_*M*_ from the fluid simulation for a monochromatic pump. (inset) seed and pump snapshots at *t* = 136 ps, showing pump depletion.

**Table 1:** Simulation output summary.

Type	Temperature [eV]	Pump chirp [Δ*ω*/*ω*]	Max. intensity [W cm^−2^]	Amplification	Duration [fs]
fluid	0	monochromatic	8.3 × 10^16^	402	40
fluid	25	monochromatic	7.8 × 10^16^	407	42
fluid	50	monochromatic	7 × 10^16^	402	45
fluid	0	3.8%	6.5 × 10^16^	334	43
fluid	25	3.8%	5.6 × 10^16^	324	47
aPIC	5 → 65 (85)	monochromatic	5.5 × 10^16^ (2.8 × 10^17^)	255 (575)	47 (15)
aPIC	5 → 65 (85)	1%	5 × 10^16^ (2.2 × 10^17^)	238 (536)	48 (19)
fluid	50	3.8%	4.7 × 10^16^	304	53
aPIC	5 → 65 (85)	3.8%	3 × 10^16^ (8.8 × 10^16^)	164 (335)	55 (40)

Seed characteristics extracted after an interaction duration *t* ≈ *t*_*M*_. In brackets values at *t* = *t*_*F*_. The amplification factor represents the energy gain.
