# Supplementary material for: Chirped pulse Raman amplification in warm plasma: towards controlling saturation
Source: Sci Rep. 2015 Aug 20;5:13333. doi: 10.1038/srep13333 (PMC4542156; doi:10.1038/srep13333)
Supplement: Supplementary Information [file srep13333-s1.pdf]

Chirped pulse Raman amplification in warm plasma: towards controlling saturation

X. Yang,[1] G. Vieux,[1] E. Brunetti,[1] B. Ersfeld,[1] J. P. Farmer,[1] M. S. Hur,[2] R. C. Issac,[1] G. Raj,[1] S. M. Wiggins,[1] G. H. Welsh,[1] S. Yoffe,[1] and D. A. Jaroszynski[1]

[1] *University of Strathclyde, SUPA, Department of Physics, Glasgow G4 0NG, United Kingdom*

[2] *UNIST, Banyeon-ri 100. Ulju-gun, Ulsan 689-798, South Korea*

EXPERIMENTAL SETUP

The schematic of the experimental setup is presented in Supplementary Figure 1. The TOPS Ti:sapphire laser system operates at 800 nm with a full width half maximum (FWHM) bandwidth of ∼ 35 nm. A 90/10 beam splitter divides the positively chirped, 250 ps long laser pulse into pump and seed beams. The pump pulse is further amplified to 1 J and transported to the vacuum chamber through a delay line, which adjusts temporal overlap. The seed pulse with a minimum duration of 70 fs and an energy of up to ≈ 2 mJ is transported to a compressor, which acts as a cut-off filter. Wavelengths above 810 nm are selected so that pump and probe satisfy the Raman resonance condition. In one set of data obtained with low pump power in Fig. 1, white light generation has been used to shift the seed spectrum. In this case the seed spectrum is centered around 836 nm instead of 817 nm.

Spherical mirrors of focal length 1.5 m focus the two beams to a waist of ∼60 µm onto opposite sides of a 4 cm long, 300 µm diameter plasma-filled hydrogen discharge capillary waveguide.

WHITE LIGHT GENERATION

The schematic for white light generation is presented in Supplemtary Figure 2. After compression, the 70 fs long seed beam is focussed by a 1 m focal length lens into a 18 mm inner diameter, 1 m long, Ar-filled tube. An aperture is used to adjust the seed spot size to optimize white light emission. A large f-number has been chosen to balance self-focusing and plasma defocusing, which occurs when the focal spot radius is about 50 µm. A spectrometer is used to characterize the white light spectrum. Typical spectral and spatial properties of the seed beam are presented in Supplementary Figure 3.

ESTIMATION OF THE PLASMA TEMPERATURE INCREASE

One common feature of a large number of particle-in-cell codes is the lack of collision modeling, in particular in the case of a moving window geometry. Our initial numerical simulation results with a cold plasma were clearly not satisfactory and using a finite but constant temperature would not take into account the spatio-temporal plasma frequency change. To simulate a more realistic interaction the thermal distribution of the electrons

entering the moving window needs to be updated at each new time step. To this end, the temperature of the plasma as function of time is calculated beforehand, taking into account as the main heating mechanism inverse bremsstrahlung from electron-ion collisions. First, the inverse bremsstrahlung damping rate is numerically solved [1]:

$$\nu(t) = \frac{Ze^2\omega_p^2}{6(2\pi)^{3/2}\varepsilon_0 m v_e(t)^3}\left[\ln\left(\frac{4mv_e(t)^2}{\hbar\omega}\right) - \gamma\right], \tag{1}$$

where Ze is the ion charge, $\hbar$ is Planck's constant, m, the electron mass, ω_p and ω are the plasma and pump laser frequencies, respectively, γ is Euler's constant and $v_e(t)$ the time-dependent thermal velocity equal to $\sqrt{v_{th}(t)^2 + v_q(t)^2}$, with $v_{th}(t)$ and $v_q(t)$ the electron thermal and quiver velocities, respectively. From the value of $\nu(t)$ the thermal velocity of the electrons is updated, numerically solving

$$\frac{\partial v_{th}(t)^2}{\partial t} = \frac{c^2}{3}\left(\nu(t)|a_0(t)|^2\right), \tag{2}$$

with $a_0(t)$ the pump envelope amplitude. The temperature $T(t)$ is given by $k_B T(t) = m v_{th}(t)^2$, where k_B is the Boltzmann constant. The temperature curve as function of time is fitted by a polynom and its coefficients are passed as input parameters to the aPIC code and used by a function to update the temperature and the resulting electron thermal distribution. Because the calculation of the change in temperature is independent of the main simulation, energy is not conserved and this solution is not self-consistent. However it does provide a better approximation to the original code.

[1] Farmer, J. P., Ersfeld, B. & Jaroszynski, D. A. Raman amplification in plasma: Wavebreaking and heating effects. *Phys. Plasmas* **17**, 113301 (2010).

FIGURES

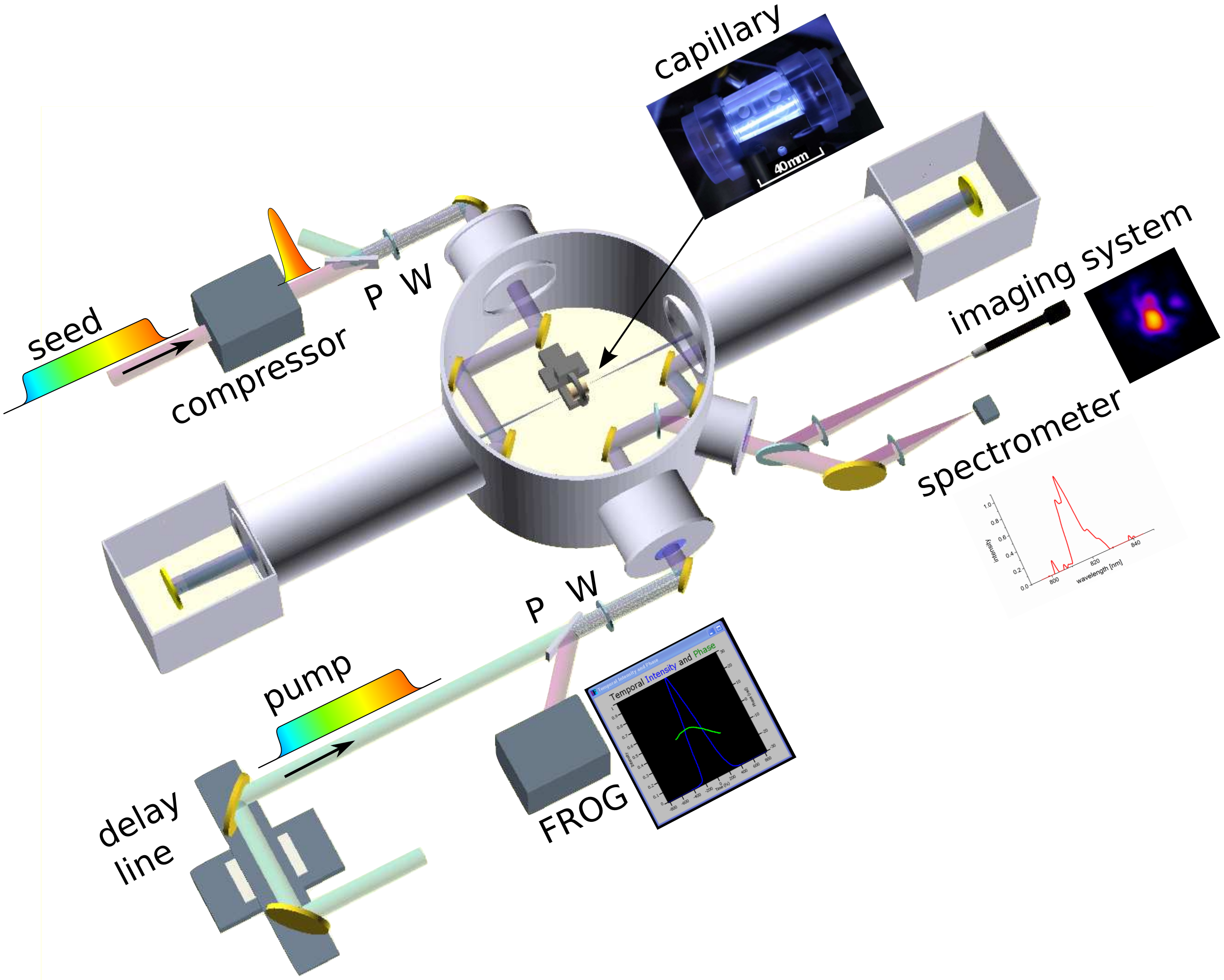

Supplementary Figure 1: Simplified schematic of the experimental layout. P and W refer to polariser and waveplate, respectively.

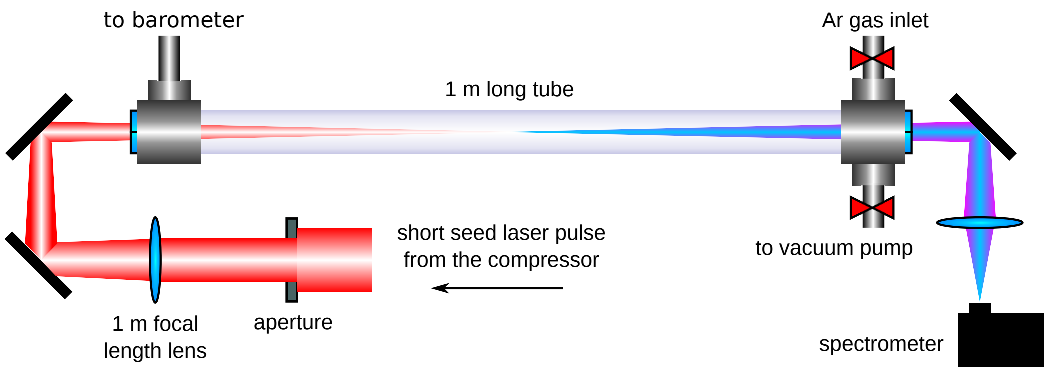

Supplementary Figure 2: White light generator.

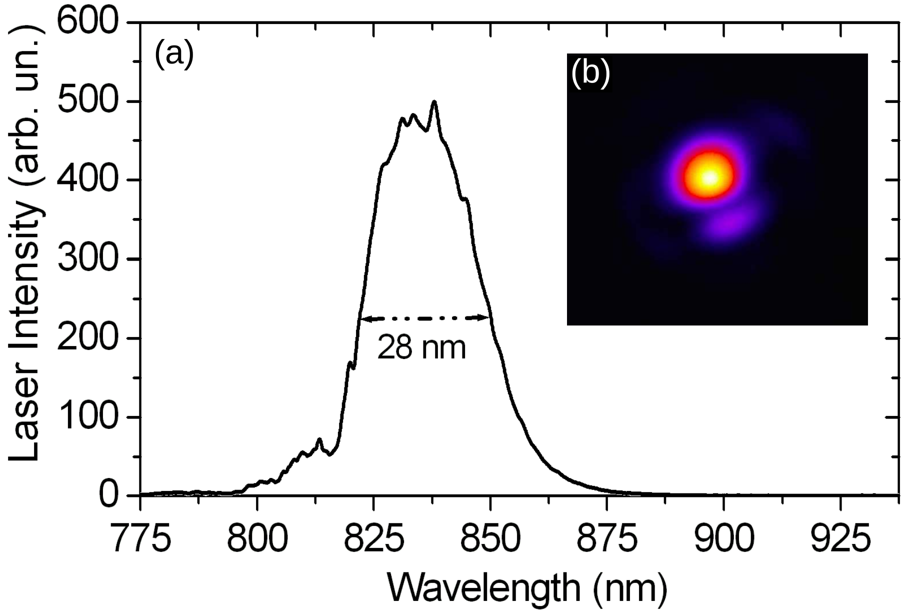

Supplementary Figure 3: (a) Spectral and (b) spatial characteristics of the seed beam after the while light generator and after filtering of the high frequency components.
